# The Theory and Practice of Medicine

**Published:** 1895-03

**Authors:** 


*The Theory and Practice of Medicine.* By FREDERICK T. ROBERTS, M.D. Ninth Edition. Pp. xvi., 1168. London: H. K. LEWIS. 1894.

The fact that this well-known and widely popular work has reached its ninth edition renders any criticism on our part superfluous. The author has kept his book well up with the progress of modern research, and it shows throughout evidence of careful revision. Two new features to which Dr. Roberts calls special notice are the increased attention that has been given to bacteriology and the sections on general therapeutics; both these add considerably to the value of the work. We have no doubt that the latest edition of this excellent text-book will meet with as much success as the former ones.

